# Anabolic Effects of a Novel Simvastatin Derivative on Treating Rat Bone Defects

**DOI:** 10.3390/biomedicines10081915

**Published:** 2022-08-08

**Authors:** Tien-Ching Lee, Hui-Ting Chen, I-Chun Tai, Li-Ting Kao, Ming-Hsin Hung, Chung-Hwan Chen, Yin-Chih Fu, Yan-Hsiung Wang, Chih-Ming Kao, Je-Ken Chang, Mei-Ling Ho

**Affiliations:** 1 Graduate Institute of Medicine, College of Medicine, Kaohsiung Medical University, Kaohsiung 807378, Taiwan; 2 Orthopaedic Research Center, College of Medicine, Kaohsiung Medical University Hospital, Kaohsiung Medical University, Kaohsiung 80708, Taiwan; 3 Regenerative Medicine and Cell Therapy Research Center, Kaohsiung Medical University, Kaohsiung 80708, Taiwan; 4 Department of Orthopedics, College of Medicine, Kaohsiung Medical University, Kaohsiung 80708, Taiwan; 5 Department of Orthopedics, Kaohsiung Municipal Ta-Tung Hospital, Kaohsiung 80145, Taiwan; 6 Department of Orthopedics, Kaohsiung Medical University Hospital, Kaohsiung Medical University, Kaohsiung 80756, Taiwan; 7 Department of Pharmacy, School of Pharmaceutical Sciences, National Yang Ming Chiao Tung University, Taipei 112304, Taiwan; 8 Department of Fragrance and Cosmetic Science, Kaohsiung Medical University, Kaohsiung 807378, Taiwan; 9 School of Pharmacy, College of Pharmacy, Kaohsiung Medical University, Kaohsiung 807378, Taiwan; 10 Reichen Biomedical Co., Ltd., Kaohsiung 804319, Taiwan; 11 School of Dentistry, College of Dental Medicine, Kaohsiung Medical University, Kaohsiung 807378, Taiwan; 12 Department of Medical Research, Kaohsiung Medical University Hospital, Kaohsiung 80756, Taiwan; 13 Department of Physiology, College of Medicine, Kaohsiung Medical University, Kaohsiung 80378, Taiwan; 14 Department of Marine Biotechnology and Resources, National Sun Yat-sen University, Kaohsiung 80424, Taiwan

**Keywords:** bone defect, bone regeneration, osteogenesis, drug, simvastatin

## Abstract

Large bone defects may develop fracture nonunion, leading to disability and psychosocial burdens. Bone grafting with anabolic agents is a good autografting alternative. Simvastatin, as a cholesterol-lowering agent worldwide, is proven to enhance osteogenesis. Considering its dose-dependent adverse effects, we developed a simvastatin derivative, named KMUHC-01, which has bone anabolic capacity and lower cytotoxicity than simvastatin. We hypothesize that KMUHC-01 could help bone formation in bone-defect animal models. We used rat models of critical calvarial and long-bone defects to evaluate the effects of KMUHC-01 and simvastatin on biological changes at the bone defect through histology, immunohistology, and mechanical testing using three-point bending and evaluated the new bone formation microstructure through microcomputed tomography analysis. The newly formed bone microstructure at the calvarial defect site showed a significantly improved trabecular bone volume in the KMUHC-01 1-μM group compared with that in the control and simvastatin groups. The biomechanical study revealed a significantly increased maximal strength in the KMUHC-01 1-μM group compared with that in the control group. KUMHC-01, as a simvastatin derivative, showed a great anabolic effect in promoting bone defect healing. However, further studies will be conducted to prove the bioavailability and bone-forming efficacy of KMUHC-01 via systemic administration.

## 1. Introduction

Removing the damaged bone and producing bone defects is often necessary after high-energy trauma, complicated fractures, bone tumors, osteonecrosis, or osteomyelitis [1]. Large bone defects may develop delayed union, malunion, and nonunion [1,2,3], which lead to high economic and social burdens [4]. Prevention and treatment of such clinical problems are challenging [5].

Bone graft substitutes (BGSs) have acceptable osteoconductive properties as a scaffold for bone growth to treat bone defects without donor site morbidity or risk of disease transmission based on bone tissue engineering development. However, they cannot be used alone for large bone defect treatment because of the limited ability of osteoinduction and osteogenesis [1,2,6]. Therefore, a combination of the BGSs with bone healing–promoting factors could be one highly potential treatment strategy that provides bone repair and regeneration elements.

Statins, 3-hydroxy-3-methylglutaryl coenzyme A reductase (HMGR) inhibitors, have been prescribed worldwide to inhibit cholesterol biosynthesis in clinical practice for more than four decades [7,8]. Recent studies have demonstrated that simvastatin plays a role in inhibiting osteoclastogenesis, enhancing osteogenesis, and promoting mineral formation in rodents [9,10,11,12,13]. Furthermore, statin-related pharmaceutics have been developed for bone fracture treatment [14,15,16]. Despite their clinical potential in osteogenesis, the effective dosage in rat models is exceptionally high [16]. Increasing the dose of statins may be accompanied by many adverse effects, such as muscle, liver, and renal damage, to achieve an effective dosage for bone formation [17,18,19].

**Figure 1:**
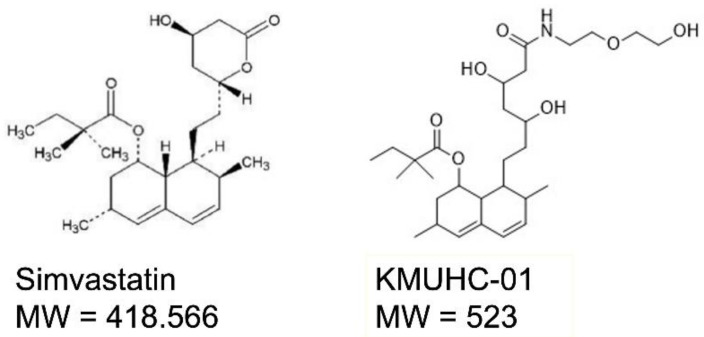
Structural formula and molecular weight (MW) of KMUHC-01 and simvastatin.

Our laboratory synthesized a novel simvastatin derivative, named KMUHC-01 (Figure 1), which has both bone selectivity and bone anabolic capacity with lower HMGR inhibition and lower cytotoxicity than simvastatin [20]. We hypothesize that KMYHC-01 is beneficial to de novo bone formation to enhance repair of bone defects. This study used a rat model of critical calvarial and long-bone defects to evaluate the biological changes in bone defect through histology, immunohistology, and mechanical testing using three-point bending and evaluated the microstructure of the newly formed bone through microcomputed tomography (micro-CT) analysis [21].

## 2. Materials and Methods

### 2.1. Cell Culture

We used mouse bone marrow mesenchymal stem cells (D1 cells), which were purchased from the American Type Culture Collection (Manassas, UA, USA) and cultured in low glucose DMEM (Gibco, Billings, MT, USA) that contained 10% fetal bovine serum (Gibco), 100 U/mL of penicillin and streptomycin (Gibco), 100 μM of MEM nonessential amino acid solution (Lonza, Basel, Switzerland), and 400 μM of L-ascorbic acid (vitamin C, Sigma, St. Louis, MO, USA) in a humidified incubator with 5% CO_2_ at 37 °C. The medium was changed every 2 days.

### 2.2. Cell Viability

Cell viability/IC50 of D1 cells treated with simvastatin/KMUHC-01 were measured using the Cell Counting Kit-8 (CCK-8, Sigma #96992) assay. CCK-8 allows sensitive and convenient assays by utilizing the highly water-soluble tetrazolium salt WST-8, which produces a water-soluble formazan dye. WST-8 is reduced by mitochondrial dehydrogenases in cells to give a yellow-colored formazan dye which is soluble in the tissue culture medium. The amount of the formazan dye generated by the dehydrogenase activity is directly proportional to the number of viable cells.

Cells were resuspended, and 200 μL of cell suspension (5 × 10^3^ cells/well) was plated in a 96-well plate. Cells were incubated in a humidified incubator with 5% CO_2_ at 37 °C for 24 h. The medium was replaced with fresh medium containing various concentrations (0.0001, 0.001, 0.01, 0.1, 1, 10, 100, and 1000 μM) of simvastatin or KMUHC-01 for 4 or 6 days. Subsequently, the CCK-8 reagent was added into wells and reach a final concentration of 10%. After 2 h incubation at 37 °C, the absorbance of samples was measured at 450 nm using a microplate reader. Cells without any treatment served as the control, and the well without cells and treatment was regarded as the blank. The percentage of cell viability was calculated using the following formula:

Cell viability (%) = (a − c)/(b − c) × 100(1)

(a = the absorbance of cells treated with various concentrations of simvastatin or KMUHC-01; b = the absorbance of the control; c = the absorbance of the blank)

The IC50 value was defined as the concentration of drug which exhibited 50% cell viability.

### 2.3. Bioactivity of Mineralization

Alizarin Red S staining was used to determine the extracellular matrix calcification level induced by KMUHC-01 in D1 cells after treatment for 1 week. In brief, D1 cells (8 × 10^4^ cells/well) were seeded in a 48-well plate. The cultures were divided into four groups as follows: control, 1 μM of simvastatin, 1 μM of KMUHC-01, and 10 μM of KMUHC-01 groups. These cells were fixed with 4% paraformaldehyde at room temperature of 25 °C for 5 min after 1 week. After washing once with double-distilled H_2_O (ddH_2_O), 300 μL of Alizarin Red S solution (2% ddH_2_O, at pH = 4.2) was added to each well in the 48-well plate. The staining solution was removed 5 min later, and each well was washed with H_2_O four times. The fixed and stained plates were then air-dried at 25 °C. The amount of mineralization was determined by dissolving the cell-bound Alizarin Red S in 10% acetic acid and spectrophotometrically quantified at 415 nm.

### 2.4. Ribonucleic Acid (RNA) Extraction and Real-Time Polymerase Chain Reaction (PCR)

Real-time PCR was utilized to analyze the osteogenic gene expression. The extraction procedure was performed according to [14]. Total RNA of the D1 cells was extracted using ZR RNA MiniPrep (Zymo Research, Irvine, CA, USA) after 12, 24, and 48 h of incubation in the osteoinduction medium. Total RNA (1 µg) was reverse-transcribed into cDNA using oligo-dT primers and a High Capacity RNA-to-cDNA Kit (Thermo Fisher Scientific, Waltham, MA, USA). Real-time PCR was performed using the Bio-Rad iQ5 real-time PCR detection system (Bio-Rad Laboratories Inc., Hercules, CA, USA) and SYBR green (Applied Biosystems, Waltham, MA, USA). The cDNA was amplified with BMP and osteocalcin (OC) primers using real-time PCR. The primers were as follows: BMP-2 forward, 5′-AGCTGCAAGAGACACCCTTTG-3′; BMP-2 reverse, 5′-AGCATGCCTTAGGGATTTTGGA-3′; OC forward, 5′-GAGGGCAATAAGGTAGTGAACA-3′; and OC reverse, 5′-AAGCCATACTGGTCTGATAGCTCG-3′. For the real-time PCR amplification, cDNA was denatured at 95 °C for 3 min, followed by 40 cycles of 95 °C for 10 s, 60 °C for 30 s, 95 °C for 5 s, and 65 °C for 5 s to generate the melting curves for the confirmation of the PCR specificity. The level of relative mRNA expression was calculated from the threshold cycle and normalized with β-actin. All real-time PCR experiments were performed three times.

### 2.5. Animals

Both 10-week-old (weight 295 ± 15 g) and 14-week-old (weight 350 ± 25 g) Sprague–Dawley male rats were purchased from Lasco Biotech and fed in the Kaohsiung Medical University (KMU) Laboratory Animal Center for 1 week before any experiments. Procedures were approved by the KMU Institutional Animal Care and Use Committee (No. 98186), and all animal experiments were conducted following the KMU guidelines for animal experiments. All animal work was conducted in an Association for Assessment and Accreditation of Laboratory Animal Care International–accredited facility. The 24 male rats that were 10 weeks old with calvarial defects were randomized into four groups (*n* = 6) as follows: a control group in which the calvarial defect was treated with phosphate-buffered saline (PBS) alone; a SIM group treated with 1 μM of simvastatin; and low HC and high HC groups treated with 0.1- and 1-μM KMUHC-01, respectively. These rats were euthanized at 4 weeks postoperatively, and then the calvarial specimens were harvested, fixed in 4% paraformaldehyde at 4 °C for 24 h, and subsequently analyzed for histomorphology. Another 24 rats that were 14 weeks old with long-bone defects were randomized into four groups (six rats per group) as follows: a control group in which the femur bone defect was treated with PBS alone; a SIM group treated with 1 μM of simvastatin; and low HC and high HC groups treated with 0.1 and 1 μM of KMUHC-01, respectively. These rats were euthanized at 6 weeks postoperatively, and then the femur bone specimens were harvested for biomechanical analysis.

### 2.6. Critical-Sized Calvarial Defects in Rats

The critical-sized calvarial defects (6 mm in width and approximately 0.7 mm in depth) were prepared with reference to our previous study [22]. The development of bone defects was under anesthesia via intraperitoneal injection of ketamine (Parke–Davis, Detroit, MI, USA; 10 mg/100 g body weight) and xylazine hydrochloride (Bayer HealthCare, Leverkusen, Germany; 12 mg/100 g body weight). The hair over the calvarium was shaved by using a depilator. A midline incision over calvarium was made and a 6 mm diameter hole was drilled to penetrate through the calvarial bone by using a trephine bur carefully with simultaneous irrigation of PBS to avoid thermal damage and damaging the dura mater. At the end of the operation, the surgical wounds were closed with 5-0 Nylon sutures. The medications (PBS, simvastatin, or KMUHC-01) were injected 3 days postoperatively into the bone defects daily for 7 days according to the grouping. All experimental rats survived and showed no wound complications throughout the experiment.

### 2.7. Femur Bone Defects in Rats

The long-bone defect model was designed according to our previous study [21]. The bone defects were developed under anesthesia as the calvarial defects model. The left thigh was shaved, and a 3 cm longitudinal skin incision was made along the lateral side. The approach was performed posterior to the vastus lateralis, and the muscle was retracted to reveal the femur bone and prevent from damaging the muscle. A bone defect of 1 × 4 mm on the left femur was developed at the middle portion of the shaft with a 0.5 mm cone-shaped bur. The bone defect was inserted with SURGICEL™ (Johnson & Johnson Medical Ltd., Neuchatel, Switzerland), and then, the drug (PBS, SIM, and KMUHC-01) was injected over SURGICEL. Skin wounds were sutured with 5-0 Nylon sutures at the end of the operation. All experimental rats survived and showed no wound complications throughout the experiment.

### 2.8. Micro-CT Analysis

We used a high-resolution microtomograph 1076 scanner (Skyscan, Bruker, Belgium) to obtain both qualitative and quantitative measurements of the bone regeneration level within the calvarial defects. The rats in the local injection design were analyzed at 0 and 4 weeks. The living rats were anesthetized intraperitoneally with ketamine/xylazine hydrochloride (1:1, 1 μL/g body weight) and bound on a holder with the sagittal axis of the cranium perpendicular to the scanning plane. The voltage and beam currents of the X-ray source were regulated at 50 kV and 200 μA, respectively, to provide the best visualization. The samples were scanned through a 360° rotation angle at 35 μm pixel size. We used Skyscan software (Bruker, Billerica, MA), including NRecon version 1.7.5.4, Skyscan CT-Analyzer program version 1.20.3.0, CT Vol: Realistic 3D-Visualization version 2.3.2.1, and Data Viewer version 1.5.6.3 to reconstruct the image data, visualize the representation scan images, and quantify the newly regenerated bone. A cylindrical region of interest (ROI) measuring 6 mm in diameter and concentric to the defect site was selected for analysis based on the CT data set. This ROI covered the original defect and the surrounding calvarial bone region. The bone growth volume was measured as the bone volume per total tissue volume.

### 2.9. Histological Analysis and Immunostaining of Bone Tissue

All bone tissue samples of the calvarial groups were decalcified (10% formic acid in ddH2O), followed by 4% paraformaldehyde fixation. The samples were then embedded in paraffin wax. The resulting 5 μm thick sections were prepared and stained with hematoxylin and eosin (H&E, Sigma-Aldrich, St. Louis, MO, USA) and Masson’s trichrome staining (TASS01, BioTnA, Kaohsiung, Taiwan). Masson’s trichrome staining was used to allow the differentiation between the remaining bone (mature–cortical) staining in red from the new bone (immature–trabecular) in blue and the nuclei in black.

H&E staining for paraffin sections was developed from the paraffinized blocks, deparaffinized in xylene, rehydrated in a graded alcohol series, and stained with hematoxylin for 10 min. Sections were stained with eosin for 3 s, dehydrated in 100% alcohol after washing for 15 min in tap water, cleared in xylene, and mounted.

The procedure for Masson’s trichrome staining was as follows: The tissue sections were prepared, deparaffinized in xylene, rehydrated, and stained with Bouin’s solution at 60 °C for 1 h. The sections were stained in Weigert’s iron hematoxylin for 10 min after washing for 10 min in tap water and then washed in tap water for 10 min and rinsed in distilled water. Next, the slides were stained in Biebrich scarlet–acid fuchsin for 10 min, rinsed in distilled water, incubated in phosphomolybdic–phosphotungstic acid for 5 min, dyed with aniline blue for 5 min, and fixed in 1% acetic acid for 2 min. Then, the slides were rinsed in distilled water, dehydrated, and mounted.

Immunohistochemistry staining for BMP-2 was performed. Briefly, tissue sections were stained with a 1:200 dilution of the BMP-2 primary antibody (Abcam, Cambridge, UK) at 4 °C overnight, followed by the HRP-conjugated secondary antibody, to observe the BMP-2 expression level using the TnAlink polymer detection system (TAHC04D, Kaohsiung, Taiwan). The sections were then counterstained with hematoxylin and were dehydrated in 100% alcohol and mounted after washing for 15 min in tap water.

The stained sections were observed with light microscopy at 40× and 100× magnification, and the images were captured with a digital camera (Nikon, Tokyo, Japan).

### 2.10. Mechanical Analysis of Femur Bone with Three-Point Bending Test

The three-point bending test was designed according to our previous study [23]. In brief, we took the rat femur and removed all the connective tissues. An Instron 4466 (Instron, Canton, MA, USA) was used for three-point bending. The harvested femur was placed onto two supports, and a single-pronged loading device was applied to the opposite surface at the midpoint between the two supports with a loading force of 1 N and speed of 1 mm/min. We measured the bone deflection at the point of load application and the load, yielding a force–deflection graph. The parameters of mechanical properties from this graph include the stiffness (the slope of the early, linear portion of the load–deflection curve), yield point, maximal load, and fracture load. Young’s modulus of the bone was estimated from the loading device geometry and the bone stiffness.

### 2.11. Statistical Analysis

All experiments were repeated at least three times, and the data (expressed as the mean ± standard error of the mean) from representative experiments are shown. Statistical significance between the groups was evaluated using one-way analysis of variance, and multiple comparisons were arranged with the Scheffe’s method. A *p*-value of <0.05 was considered statistically significant.

## 3. Results

### 3.1. KMUHC-01 Is Less Cytotoxic Than Simvastatin

**Figure 2:**
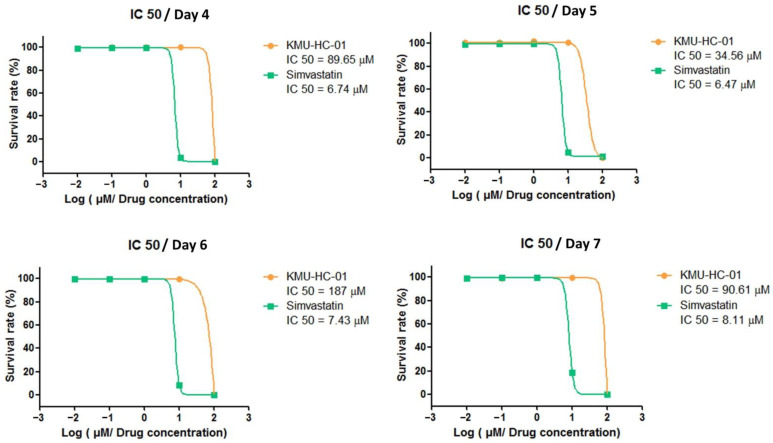
Cytotoxicity of simvastatin and KMUHC-01. Cell viability/IC50 of D1 cells treated with simvastatin/KMUHC-01 for 4 and 7 days were measured using the CCK-8 assay.

The cytotoxic effects of KMUHC-01 were measured using the CCK-8 assay. The IC50 values of the D1 cells that were treated with KMUHC-01 and simvastatin for 4 days are 89.65 and 6.74 μM, respectively, and for 7 days, 90.61 and 8.11 μM, respectively (Figure 2).

### 3.2. Both KMUHC-01 and Simvastatin Enhanced D1 Cell Mineralization and BMP-2 Expression

**Figure 3:**
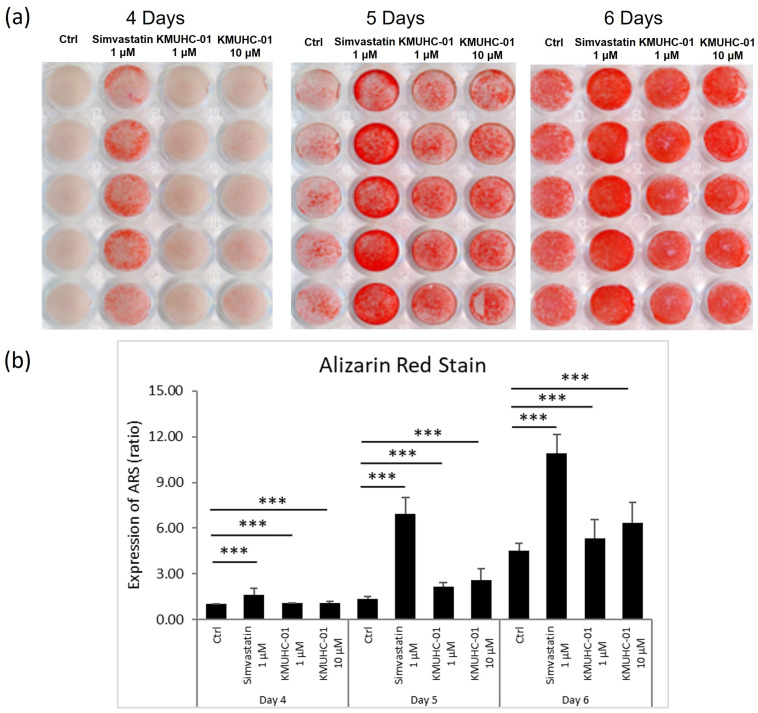
Mineralization of D1 cells treated with simvastatin and KMUHC-01. (**a**)Alizarin Red S (ARS) staining; (**b**) the quantified amount of calcified material. (*** *p* < 0.005, comparison with control group).

**Figure 4:**
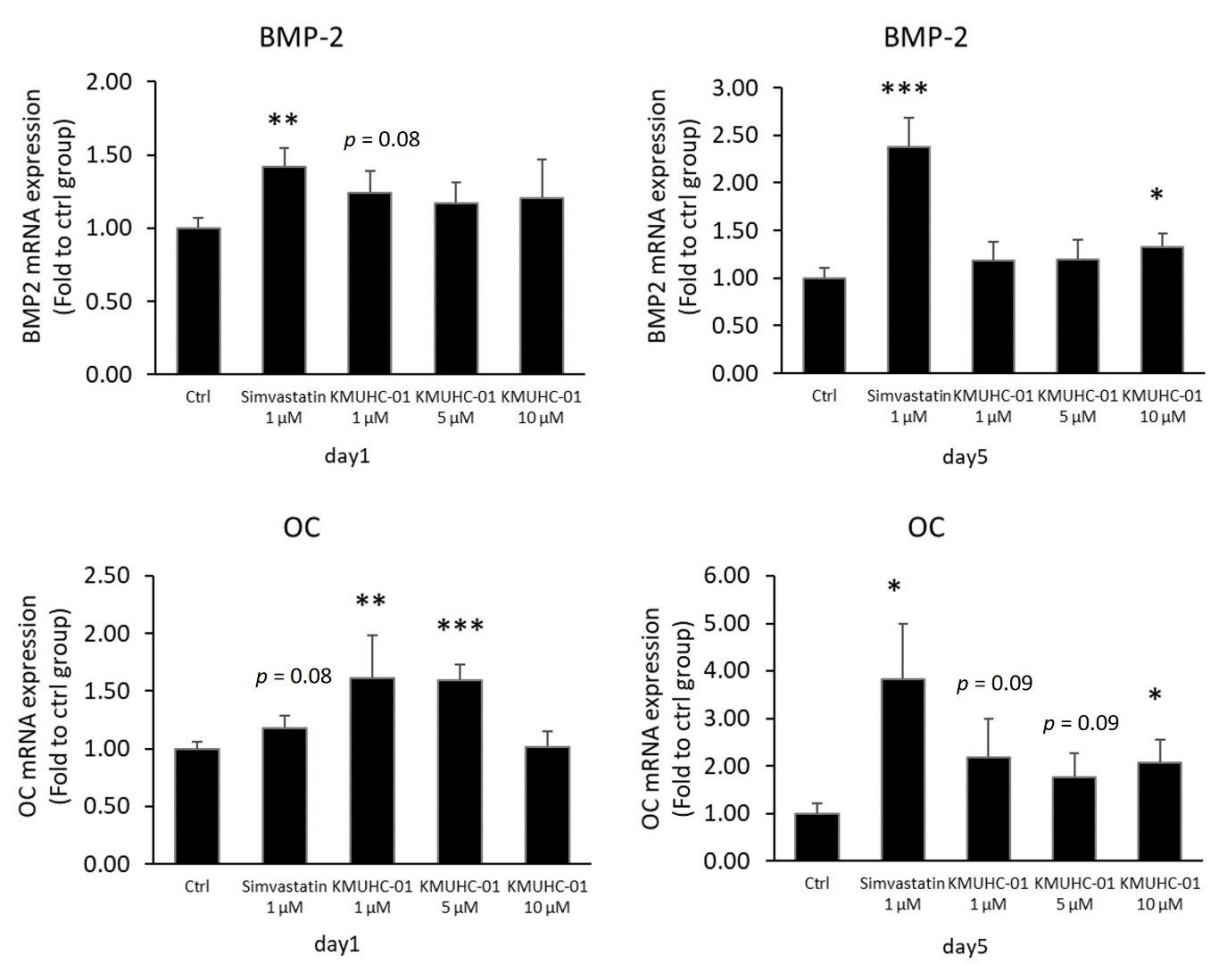
BMP-2 and osteocalcin (OC) gene expression determined using real-time quantitative polymerase chain reaction (PCR) analysis of D1 cells. (* *p* < 0.05; ** *p* < 0.01; *** *p* < 0.001, comparison with control group).

The quantitative results from Alizarin Red staining showed that the simvastatin 1 μM, KMUHC-01 1-μM and 10-μM groups had significantly enhanced D1 cell mineralization compared with the control group. Further, the effect on the induction of mineralization in the simvastatin 1 μM group was better than that in the KMUHC-01 1 μM and 10 μM groups (Figure 3a,b). We measured the mRNA levels of osteogenic genes (BMP-2 and OC) in the D1 cells treated with simvastatin and KMUHC-01 (Figure 4) and found that the simvastatin 1 μM and KMUHC-01 10 μM groups showed more BMP2 gene expression than the control group, with significance, on day 5. Other groups of KMUHC-01 showed increased BMP-2 expression without statistical significance. The KMUHC-01 1 and 5 μM groups showed a significantly increased OC expression compared with the control group on day 1. The simvastatin 1 μM and KMUHC-01 10 μM groups showed significantly increased OC expression by day 5.

### 3.3. KMUHC-01 and Simvastatin Treatments Enhance Bone Formation Evidenced by Micro-CT Analysis, H&E, and Masson’s Trichrome Staining of Critical-Sized Calvarial Defects

**Figure 5:**
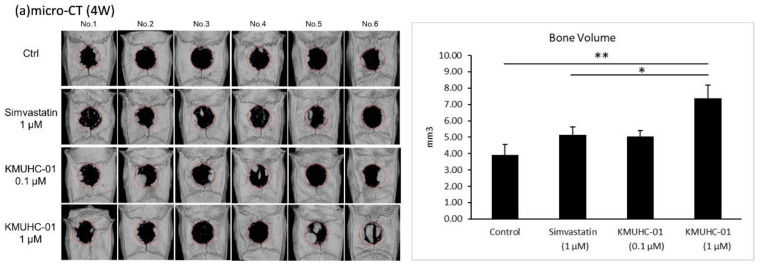

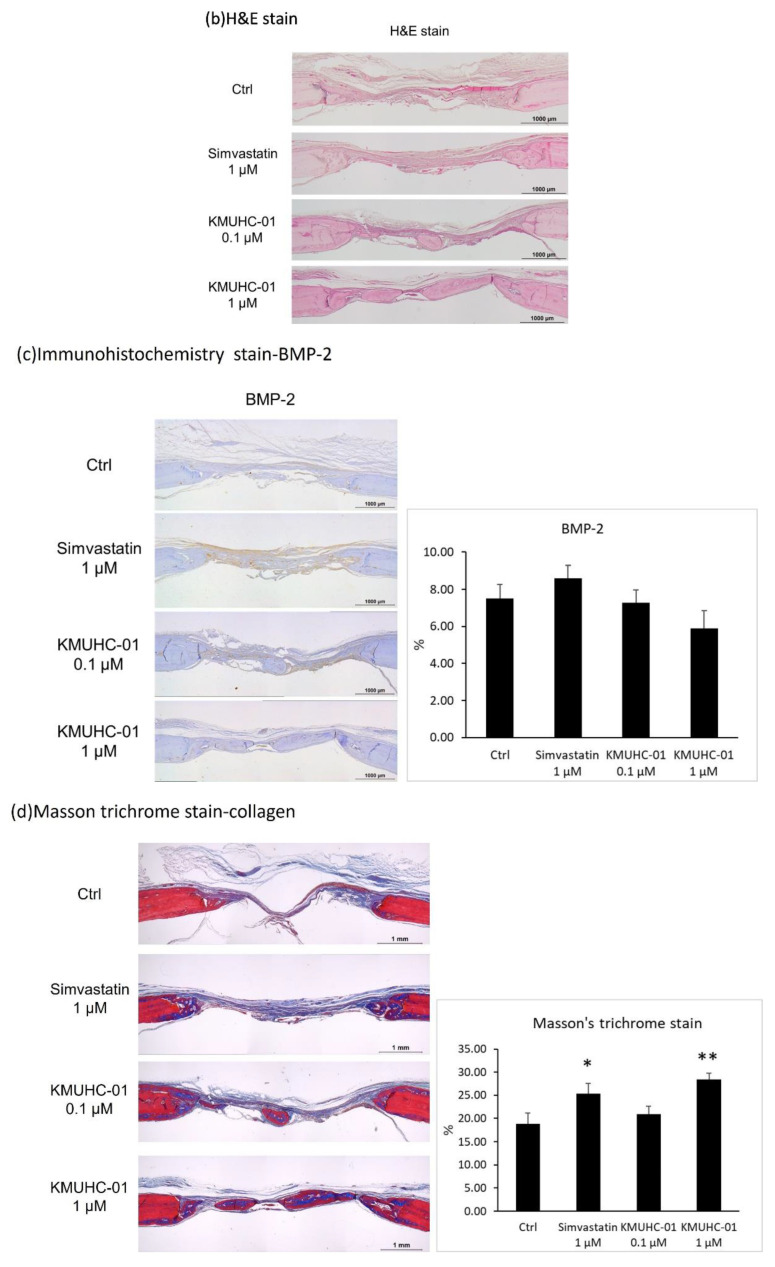
Rat calvarial bone defect treated with local simvastatin and KMUHC-01 injections. (**a**) The radiography study with micro-CT and the data of bone volume within the bone defect at 4 weeks postoperatively; (**b**) the representative sections of the histological study with hematoxylin and eosin (H&E) staining; (**c**) immunohistochemistry staining for BMP-2; and (**d**) Masson’s trichrome staining. (* *p* < 0.05; ** *p* < 0.01, comparison with control group).

Representative photographs of the 3D, reconstructed micro-CT scans of the rat calvarial defects are shown in Figure 5a. The high HC group displayed significantly more bone regeneration than the control and SIM groups at 4 weeks.

The rat calvaria were harvested and analyzed using H&E and Masson’s trichrome staining to evaluate the histologic effect of KMUHC-01 and simvastatin on bone repair. The high HC group showed obvious new bone formation in the calvarial defects; the other three groups exhibited mostly fibrous tissue with minimal bone formation at 4 weeks after injection (Figure 5b), which is similar to the micro-CT results. The blue color in Masson’s trichrome staining indicates the regenerated bone, collagen fibers, or osteoid. There is significantly more blue staining in the SIM and high-HC groups than in the control group (Figure 5d).

### 3.4. Immunohistochemistry Staining of BMP-2 Critical-Sized Calvarial Defects

Immunohistochemistry was used to examine BMP-2 expression in a newly formed bone at 4 weeks postoperatively (Figure 5c). There was no significant difference among these groups although the SIM group showed mildly more BMP-2 staining.

### 3.5. KMUHC-01 Could Help Recover the Mechanical Properties of Femur Bone Defect

**Figure 6:**
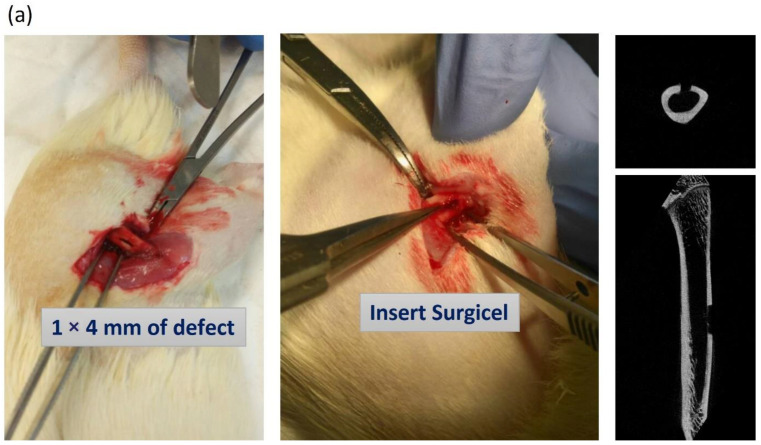

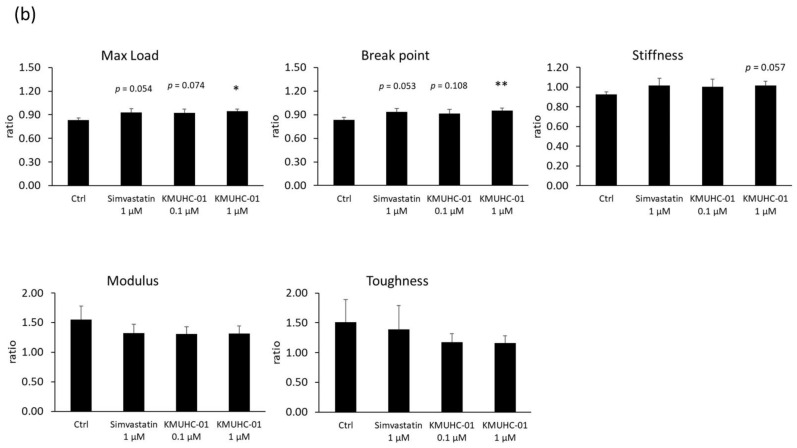
Rat femur bone defect treated with local simvastatin and KMUHC-01 injections. (**a**) A representative rat femur bone defect model is shown in the experimental photograph and the image of micro-CT postoperatively; (**b**) the biomechanics study of three-point bending tests to analyze the mechanical properties at the end of femur bone defect model (6 weeks postoperatively), which are expressed using the defect-to-nondefect femur ratio. (* *p* < 0.05; ** *p* < 0.01, comparison with control group).

The three-point bending test was employed to analyze the mechanical properties at the end of the femur bone defect model. (Figure 6a,b). The contralateral femoral bone served as a nondefect femoral bone. The defect-to-nondefect femur ratios were used to express the biomechanical study data. The raw data of the bending test are shown in Table S1. The three-point bending test results show that the maximal load ratio in the high-HC group was significantly higher than that in the control group 6 weeks postoperatively (*p* = 0.029). The breakpoint ratio in the high-HC group was also higher than that in the control group (*p* = 0.027). There was no significant difference in the total energy absorption (toughness) and Young’s modulus (stiffness) ratio among these four groups.

## 4. Discussion

The present study revealed that KMUHC-01 has lower in vitro cell toxicity than simvastatin. The high-HC group showed anabolic effects in vitro and in vivo, which were proven using the rat calvarial bone defect model, a model of de novo bone formation. The microstructure of the newly formed bone at the calvarial defect site showed that the high-HC group significantly improved the trabecular bone volume compared with the control and SIM groups. The biomechanical study results showed that the maximal strength was significantly increased in the high-HC group compared with the control group.

The mineralization study showed that the effect of the simvastatin 1 μM group was better than that of the KMUHC-01 0.1 μM and 10 μM groups (Figure 3a,b). The results are comparable with the study of BMP-2 expression of D1 cells, in which the number of cells treated with KMUHC-01 at 1 μM was not more than that of the D1 cells treated with simvastatin at 1 μM on days 1 and 5 according to the real-time PCR data of osteogenic gene expression. However, the performance of the high-HC group in de novo bone formation in calvarial and femur defects was better than that of the SIM group. The inconsistency may result from the different timing of osteogenic gene expression stimulation or a different anabolic effect mechanism. Therefore, further study is being pursued.

The positive effects of simvastatin on bone formation were first proven by Mundy et al. in 1999 [23], and further studies have demonstrated that simvastatin acts as an osteoclastogenesis inhibitor and osteogenesis enhancer and promotes mineral formation in rodents [9,10,11,12,13]. Their effects are considered related to other product reductions that originate from the mevalonic acid pathway, including farnesyl pyrophosphate (FPP) and geranylgeranyl pyrophosphate (GGPP), which are isoprenoid precursors [24]. Maeda et al. demonstrated that simvastatin can increase osteoblastogenesis by inducing the expression of BMP-2, alkaline phosphatase, OC, and vascular endothelium growth factor [25]. Similarly, Weivoda et al. revealed that statins enhance osteoblastic differentiation and their bone matrix mineralization by inhibiting FPP and GGPP syntheses [26]. However, the statin-induced bone formation mechanism may involve alternative pathways. Li and Chuang et al. revealed that simvastatin can enhance estrogen receptor alpha (ERα) expression by acting as an ERα ligand and coactivator to promote bone formation [27,28]. Furthermore, Hsieh et al. demonstrated that some novel simvastatin derivatives without HMGR inhibition activity have an anabolic effect on bone formation [20]. The present study proved that the local application of KMUHC-01 can enhance bone defect repair with rat calvarial and femur defect models. There is a good possibility that KMUHC-01 has the potential to be an anabolic agent to promote bone repair.

This study had limitations. First, KMUHC-01 is a simvastatin derivative, but KMUHC-01 and simvastatin have different osteogenesis-related gene expression patterns. Additionally, the detailed KMUHC-01 mechanism in bone repair remains not well known. Second, the case number and follow-up duration were limited. The adverse effect may be dose or time dependent. Therefore, further studies are needed to discover the positive and adverse effects of KMUHC-01 although this study revealed low cytotoxicity.

## 5. Conclusions

We used calvarial and femoral long-bone defect models to reveal the anabolic effect of local KMUHC-01 and revealed that KMUHC-01 enhanced the BMP-2 protein level in repaired bone and accelerated de novo bone formation while increasing mechanical properties in the bone defect model. KUMHC-01, as a simvastatin derivative without HMGR affinity, showed a great anabolic effect on promoting bone defect healing in the present study. Therefore, further studies are necessary to prove the bioavailability and bone-forming efficacy of KMUHC-01 via systemic administration.

## 6. Patents

This section is not mandatory but may be added if there are patents resulting from the work reported in this manuscript.

## Data Availability

The data that support the findings of this study are not publicly available but can be accessed with permission from the Kaohsiung Medical University Hospital in Taiwan.

## Supplementary Materials

The following are available online at https://www.mdpi.com/article/10.3390/biomedicines10081915/s1, Table S1: The biomechanics study of the three-point bending test.
