# Supplementary material for: Anabolic Effects of a Novel Simvastatin Derivative on Treating Rat Bone Defects
Source: Biomedicines. 2022 Aug 8;10(8):1915. doi: 10.3390/biomedicines10081915 (PMC9405916; doi:10.3390/biomedicines10081915)
Supplement: Supplementary file 1 [file biomedicines-10-01915-s001.zip › biomedicines-1804707-supplementary.pdf]

Table S1: The biomechanics study of the three-point bending test.

The biomechanic study of three-point bending test

Max Load	Mean ± SD			p value
	Left	Right	Ratio	
Ctrl	152.349 ± 2.646	183.708 ± 3.862	0.831 ± 0.030	-
Simvastatin 1 µM	164.122 ± 10.522	177.977 ± 12.251	0.931 ± 0.047	0.054
KMUHC-01 0.1 µM	159.521 ± 5.564	175.184 ± 8.591	0.924 ± 0.051	0.074
KMUHC-01 1 µM	158.187 ± 5.192	167.793 ± 4.904	0.945 ± 0.027	0.012

Break point	Mean ± SD			p value
	Left	Right	Ratio	
Ctrl	152.349 ± 2.646	182.617 ± 3.165	0.836 ± 0.028	-
Simvastatin 1 µM	164.122 ± 10.522	177.468 ± 12.040	0.933 ± 0.046	0.053
KMUHC-01 0.1 µM	157.483 ± 5.437	174.899 ± 8.475	0.914 ± 0.052	0.108
KMUHC-01 1 µM	158.187 ± 5.192	166.577 ± 5.671	0.953 ± 0.029	0.009

Stiffness	Mean ± SD			p value
	Left	Right	Ratio	
Ctrl	320.228 ± 13.352	346.790 ± 15.432	0.925 ± 0.025	-
Simvastatin 1 µM	347.648 ± 24.465	350.865 ± 33.572	1.018 ± 0.070	0.124
KMUHC-01 0.1 µM	373.817 ± 7.297	391.678 ± 37.905	1.003 ± 0.077	0.18
KMUHC-01 1 µM	325.985 ± 13.100	328.123 ± 25.906	1.014 ± 0.045	0.057

Modulus	Mean ± SD			p value
	Left	Right	Ratio	
Ctrl	23.135 ± 0.686	16.513 ± 3.614	1.549 ± 0.230	-
Simvastatin 1 µM	21.587 ± 1.156	17.611 ± 2.056	1.322 ± 0.147	0.22
KMUHC-01 0.1 µM	25.851 ± 2.078	21.229 ± 3.011	1.304 ± 0.124	0.197
KMUHC-01 1 µM	22.237 ± 1.588	17.800 ± 1.689	1.312 ± 0.129	0.205

Toughness	Mean ± SD			p value
	Left	Right	Ratio	
Ctrl	0.768 ± 0.112	0.582 ± 0.113	1.511 ± 0.376	-
Simvastatin 1 µM	0.734 ± 0.043	0.691 ± 0.111	1.386 ± 0.404	0.413
KMUHC-01 0.1 µM	0.720 ± 0.103	0.609 ± 0.055	1.175 ± 0.138	0.226
KMUHC-01 1 µM	0.650 ± 0.058	0.576 ± 0.038	1.156 ± 0.122	0.212
